# The effect of everolimus on renal angiomyolipoma in pediatric patients with tuberous sclerosis being treated for subependymal giant cell astrocytoma

**DOI:** 10.1007/s00467-017-3806-1

**Published:** 2017-10-09

**Authors:** John J. Bissler, David N. Franz, Michael D. Frost, Elena Belousova, E. Martina Bebin, Steven Sparagana, Noah Berkowitz, Antonia Ridolfi, J. Christopher Kingswood

**Affiliations:** 1 Division of Nephrology, St Jude Children’s Research Hospital and Le Bonheur Children’s Hospital, University of Tennessee Health Science Center, 49 North Dunlap Street, Memphis, TN 38163 USA; 2 Cincinnati Children’s Hospital Medical Center, Cincinnati, OH USA; 3 Minnesota Epilepsy Group, St Paul, MN USA; 4 Moscow Research Institute of Pediatrics and Pediatric Surgery, Moscow, Russia; 5 University of Alabama School of Medicine, Birmingham, AL USA; 6 Texas Scottish Rite Hospital for Children, Dallas, TX USA; 7 Novartis Pharmaceuticals Corporation, East Hanover, NJ USA; 8 Novartis Pharmaceuticals S.A.S., Rueil-Malmaison, France; 9 Royal Sussex County Hospital, Brighton, UK

**Keywords:** Angiomyolipoma, Everolimus, Pediatrics, Subependymal giant cell astrocytoma, Tuberous sclerosis complex

## Abstract

**Background:**

Patients with tuberous sclerosis complex (TSC) often have multiple TSC-associated hamartomas, particularly in the brain and kidney.

**Methods:**

This was a post hoc analysis of pediatric patients being treated for subependymal giant cell astrocytomas (SEGAs) during the phase 3, randomized, double-blind, placebo-controlled EXIST-1 trial. Patients were initially randomly assigned to receive everolimus 4.5 mg/m^2^/day (target blood trough 5–15 mg/dl) or placebo and could continue in an open-label extension phase. Angiomyolipoma response rates were analyzed in patients aged <18 years with ≥1 target angiomyolipoma lesion at baseline. Response was defined as the proportion of patients with a ≥50% reduction in the sum volume of target renal angiomyolipomata from baseline, in the absence of new target angiomyolipomata, a >20% increase in kidney volume from nadir, and angiomyolipoma-related bleeding ≥ grade 2. Tolerability was also assessed.

**Results:**

Overall, this analysis included 33 patients. Renal angiomyolipoma response was achieved by 75.8% of patients (95% confidence interval, 57.7–88.9%), with sustained mean reductions in renal angiomyolipoma volume over nearly 4 years of treatment. In addition, most (≥80%) achieved clinically relevant reductions in angiomyolipoma volume (≥50%), beginning at week 24 and continuing for the remainder of the study. Everolimus was generally well tolerated in this subgroup, with most adverse events being grade 1 or 2 in severity.

**Conclusions:**

Although everolimus is currently not indicated for this use, this analysis from EXIST-1 demonstrates its long-term efficacy and safety for the treatment of renal angiomyolipoma in pediatric patients undergoing treatment for TSC-associated SEGA.

**Electronic supplementary material:**

The online version of this article (10.1007/s00467-017-3806-1) contains supplementary material, which is available to authorized users

## Introduction

Tuberous sclerosis complex (TSC) is an autosomal dominant hereditary disorder occurring in 1 in 5,800 births and affecting approximately 1 million individuals worldwide [1, 2]. This genetic disorder is caused by mutations in either the *TSC1* or the *TSC2* gene, which results in aberrant activation of the mammalian target of the rapamycin complex 1 (mTORC1) signaling pathway [3]. Increased mTORC1 signaling results in growth of hamartomas, which may begin in utero, in several organs throughout the body, including the kidneys, skin, brain, liver, lungs, and heart [4, 5].

Although classified as benign, TSC-associated tumors may have significant negative impacts on organ function. Many patients with TSC exhibit cortical tubers and develop subependymal nodules (SEN) in the brain prenatally or early in life, which can continue growth as slow-growing glioneural tumors known as subependymal giant cell astrocytomas (SEGAs), which may lead to serious complications such as acute hydrocephalus and death [6–8]. Renal angiomyolipomata are a common TSC-related manifestation, occurring in up to 80% of patients [9]. These lesions typically occur bilaterally, and patients often present with multiple tumors in each kidney that comprise smooth muscle-like cells, abnormal blood vessels, and adipose-like cells [10]. Renal angiomyolipomata, often identified by adolescence, grow with age [11, 12]. Angiomyolipoma size (>3 cm in the longest diameter) and serial growth are risk factors for complications such as developing aneurysms that can rupture, resulting in hemorrhage [13, 14]. Growing angiomyolipomata also have the potential to slowly compress or infiltrate healthy renal tissue, compromising function and increasing the risk for renal failure [15]. As a result, they are a significant cause of morbidity and mortality in patients with TSC, highlighting the need for early detection and treatment [9, 16, 17].

Everolimus, an oral mTORC1 inhibitor, was assessed as a treatment for renal angiomyolipoma in the phase 3, double-blind, placebo-controlled study EXamining everolimus In a Study of Tuberous sclerosis complex (EXIST-2; NCT00790400). Based on the superior response rate for everolimus versus placebo (42% vs 0%; *p* < 0.0001; after a median duration of treatment of 38 weeks for everolimus and 34 weeks for placebo) [18], everolimus was approved for the treatment of TSC-associated angiomyolipoma in adult patients. More recently, longer-term interim analysis of the data demonstrated a further improvement in response rate to 54% after approximately 2.5 years during an extension phase of EXIST-2 [19]. In a prior study, EXIST-1 (NCT00789828), a phase 3, a double-blind, placebo-controlled study in patients with TSC-associated SEGAs, treatment with everolimus had achieved a superior SEGA response rate compared with placebo during the double-blind core phase (35% vs 0%; *p* < 0.0001; after a median duration of treatment of 42 weeks for everolimus and 36 weeks for placebo) [20]. This led to the approval of everolimus for the treatment of SEGA in adult and pediatric patients, its first TSC indication. Everolimus is currently not approved to treat TSC-associated angiomyolipomata in pediatric patients owing to a lack of data in this subpopulation [21].

Many patients with TSC enrolled in EXIST-1 also had angiomyolipoma, making possible an evaluation of the effect of everolimus on angiomyolipoma in pediatric patients. The median age of patients who received at least one dose of everolimus in EXIST-1 was 9.5 years (range 1.1–27.4 years) [22]. The long-term extension phase of EXIST-1 was recently concluded, and the final analysis reported sustained efficacy in SEGA reduction for approximately 4 years of treatment, with 57.7% of patients achieving a SEGA response at any time [23]. This confirmed a durable response to everolimus for the indicated treatment of pediatric and adult patients with TSC-related SEGA [21]. Renal angiomyolipoma response rate was a predefined exploratory end point of EXIST-1 in the subset of patients with ≥1 target angiomyolipoma (longest lesion diameter ≥1.0 cm) at baseline [20, 24]. An angiomyolipoma response rate of 53% was observed in the everolimus arm compared with a response rate of 0% in the placebo arm during the double-blind core phase in patients primarily aged <18 years [20, 24]. Here, we present a post hoc analysis of longer-term renal angiomyolipoma response and tolerability data in these pediatric patients at the conclusion of the EXIST-1 study.

## Materials and methods

The methods for the EXIST-1 study have been previously described [20, 22-24]. In brief, patients of any age with a diagnosis of TSC and serial SEGA growth were randomly assigned 2:1 to receive everolimus or placebo in the primary core phase of the study. Everolimus was orally administered and initiated at a dose of 4.5 mg/m^2^ body surface area per day and subsequently titrated to blood trough levels of 5–15 ng/ml subject to tolerability. The primary end point of the study was SEGA response rate, defined as the proportion of patients with a ≥50% reduction in the sum SEGA volumes relative to baseline, with no worsening of nontarget SEGA lesions, no new SEGA lesions (≥1 cm in the longest diameter), and no new or worsening hydrocephalus.

After achieving positive results during the core phase (time from the start of the trial to the time when the last patient had received study treatment for 6 months), the study continued with a preplanned open-label extension phase in which all patients remaining in the study could receive everolimus. The extension phase continued until 4 years after the last patient had been randomly assigned.

The study protocol was approved by an ethics committee at each center, before the first patient was enrolled. The study was conducted in accordance with the principles of good clinical practice, the Declaration of Helsinki, and all local regulations. All patients (or their legal representatives) provided written informed consent before enrollment.

### Study end points and assessments

This post hoc analysis assessed the renal angiomyolipoma response rate in patients with 1 or more target angiomyolipomas (longest lesion diameter ≥1.0 cm), which was a preplanned exploratory end point of EXIST-1. As in EXIST-2, the renal angiomyolipoma response rate was defined as the proportion of patients with a ≥50% reduction in the sum of target renal angiomyolipoma volume relative to baseline based on independent central radiology review, with no new target angiomyolipoma, no increase in kidney volume of >20% from nadir, and no angiomyolipoma-related bleeding of grade 2 or higher (based on the National Cancer Institute Common Terminology Criteria for Adverse Events [NCI-CTCAE], version 3.0 [25]).

Tumor volume was measured by computed tomography or magnetic resonance imaging (MRI) assessment of the kidney, which was performed 12, 24, and 48 weeks after the start of treatment and annually thereafter, and required a confirmation scan approximately 12 weeks after the initial response was observed. The same imaging modality was to be used throughout the trial for each patient. Mean volume reduction was calculated, in addition to the proportion of patients with ≥30% or ≥50% reductions in the sum of volumes of target angiomyolipomata. A waterfall plot was constructed showing the best volume reduction achieved by each patient at any time point.

Estimated glomerular filtration rate (eGFR), calculated using the Schwartz formula [26], was assessed at baseline, at weeks 2, 4, 6, 8, 12, and 18, then every 12 weeks thereafter. Furthermore, the assessment of protein urinalysis was conducted by urine dipstick at baseline, weeks 4, 8, 12, 18, and 24, and every 12 weeks thereafter.

Adverse events (AEs) were monitored continuously throughout the study and were graded according to NCI-CTCAE (version 3.0) [25].

### Statistical analyses

All analyses were performed on pediatric patients from the EXIST-1 trial (i.e., <18 years of age at the start of treatment) who received ≥1 dose of everolimus and had ≥1 target renal angiomyolipoma at baseline. Exact 95% confidence intervals (CIs) for renal angiomyolipoma response rates were obtained using the Clopper–Pearson method. Statistical analyses were performed using SAS software (version 9.2). The data cutoff date was the day of study completion (2 October 2014).

## Results

Of the 117 patients initially randomly assigned in the double-blind phase, 111 received ≥1 dose of everolimus, either as randomized treatment or as an open-label treatment (33 patients had originally been assigned to placebo and switched to everolimus). Among the patients who received everolimus, 33 were <18 years of age at the start of treatment and had ≥1 target angiomyolipoma at baseline. This pediatric subgroup was included in the current analysis, with 23 of these patients (69.7%) completing the study per protocol. Reasons for early discontinuation included AEs (*n* = 3; 9.1%), patient withdrawal of consent (*n* = 3; 9.1%), administrative problems (*n* = 3; 9.1%), and loss to follow-up (*n* = 1; 3.0%).

More than half (54.5%) of the patients in this subgroup were male, and most were white (90.9%; Table 1). The median (range) age of these patients was 11.5 (5.4–17.5) years, with 39.4% aged between 3 and <10 years (Table 1). Most (81.8%) patients had a lesion size of <3 cm at baseline. At the time of study completion (2 October 2014), median duration of everolimus exposure in these patients was 44.8 months (range 1.9–57.9). The mean everolimus trough concentration (C_min_) increased over the first 6 weeks from 4.40 ng/ml at week 2, to 5.09 ng/mL at week 4, and 5.76 ng/ml at week 6, and then remained at approximately between 6 and 8 ng/ml for the remainder of the study. All but one patient (32 out of 33) had taken antiepileptic medications during the study.

**Table 1:** Baseline demographics of patients aged <18 years with renal angiomyolipoma

	Everolimus (*n* = 33)
Age (years)	
Mean (SD)	11.5 (3.54)
Median (range)	11.5 (5.4–17.5)
Age category	
3 to <10 years	13 (39.4)
10 to <18 years	20 (60.6)
Male	18 (54.5)
Race	
White	30 (90.9)
Black	2 (6.1)
Other	1 (3.0)
Lesion size^a^	
<3 cm	27 (81.8)
≥3 cm	6 (18.2)
Body surface area (m^2^)	
Mean (SD)	1.32 (0.38)
Median (range)	1.28 (0.8–2.2)

Data are presented as the number of patients, with the percentage given in parenthesis, the mean ± standard deviation (SD), or the median, with the range given in parenthesis, as appropriate

^a^Lesion size was measured by the maximum major axis axial or all-plane diameter of the largest lesion

### Efficacy

**Fig. 1:**
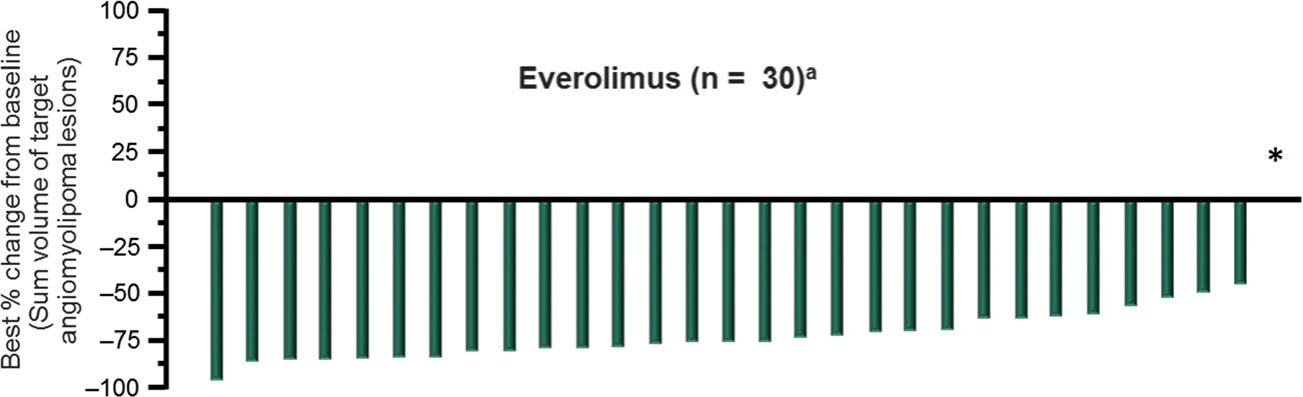
Best percentage change in renal angiomyolipoma volume on treatment. *Percentage change in sum of volumes of target angiomyolipoma lesion available for one subject, but contradicted by overall angiomyolipoma response = progressive disease. ^a^Patients for whom best percentage change in target angiomyolipoma lesion volume was not available or with overall response of “not evaluable” were excluded from the graph

**Fig. 2:**
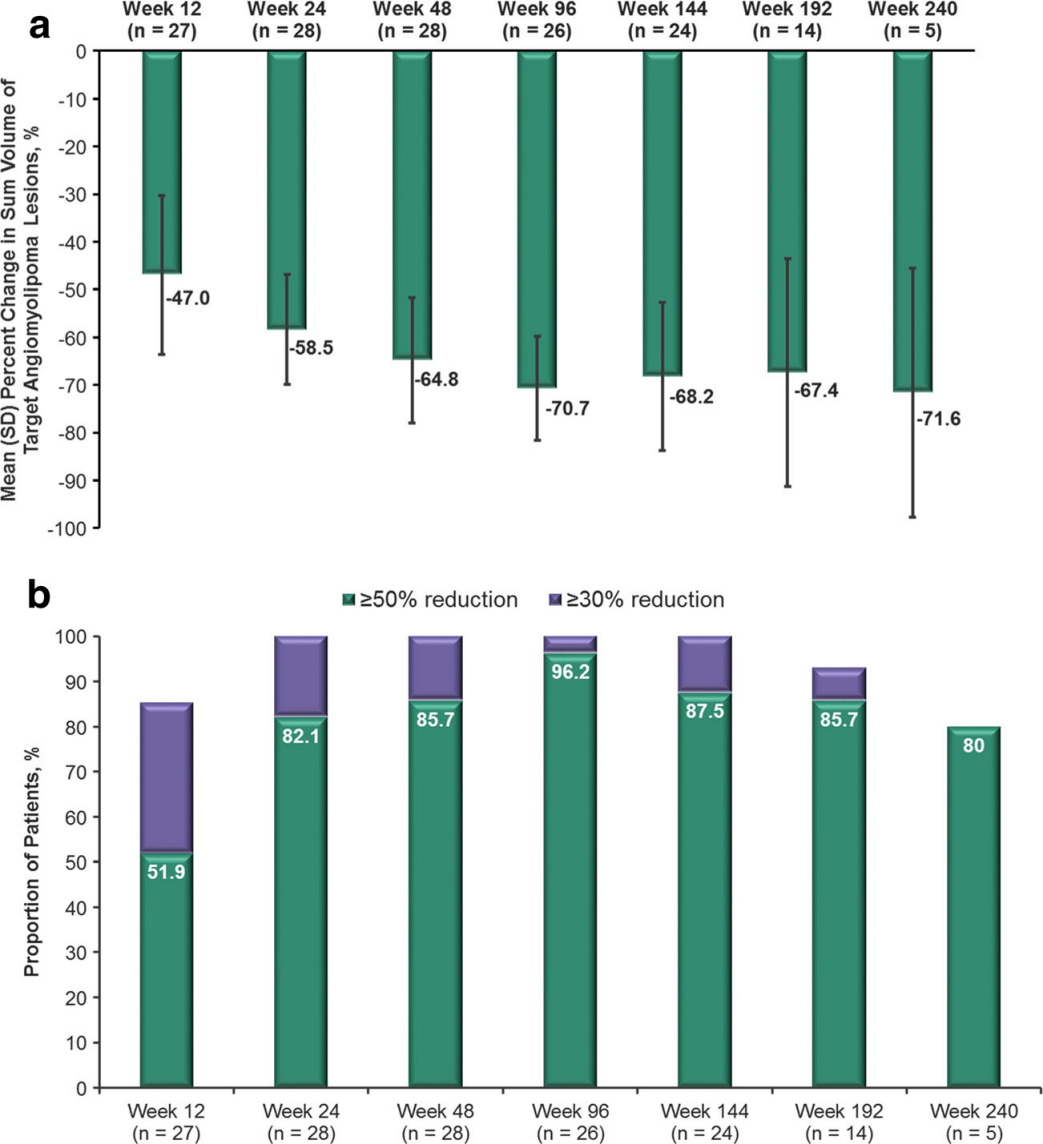
**a** Mean (standard deviation) reductions in the sum of volume of target angiomyolipoma lesions over time and** b** the proportions of patients achieving an angiomyolipoma volume reduction of ≥50% or ≥30% over time

Among the 33 patients with angiomyolipoma at baseline, a renal angiomyolipoma response was reported in 25 patients (75.8%, 95% CI 57.7–88.9%) and stable disease was reported as a best response in 4 patients (12.1%). Best percentage change from baseline could be determined for 30 patients (Fig. 1). Of these, 29 (96.7%) had a reduction in their renal angiomyolipoma volume relative to baseline as their best response. The mean percentage reduction of renal angiomyolipoma volume improved from 47% at week 12 to 70.7% at week 96, and then stabilized for the duration of the study (Fig. 2a), remaining above 67% through week 240.

Most patients (≥80%) experienced clinically relevant reductions in angiomyolipoma volume (≥50%) from week 24 through the remainder of the study (Fig. 2b). At week 192 (*n* = 14), 92.9% had ≥30% reduction in renal angiomyolipoma volume, and 85.7% had ≥50% reduction in volume, demonstrating that clinically relevant reductions were sustained over time. An additional patient had a reduction of ≥30% at week 144 (30.5% reduction from baseline), but did not meet this cutoff at week 192 (0.81% reduction from baseline).

**Fig. 3:**
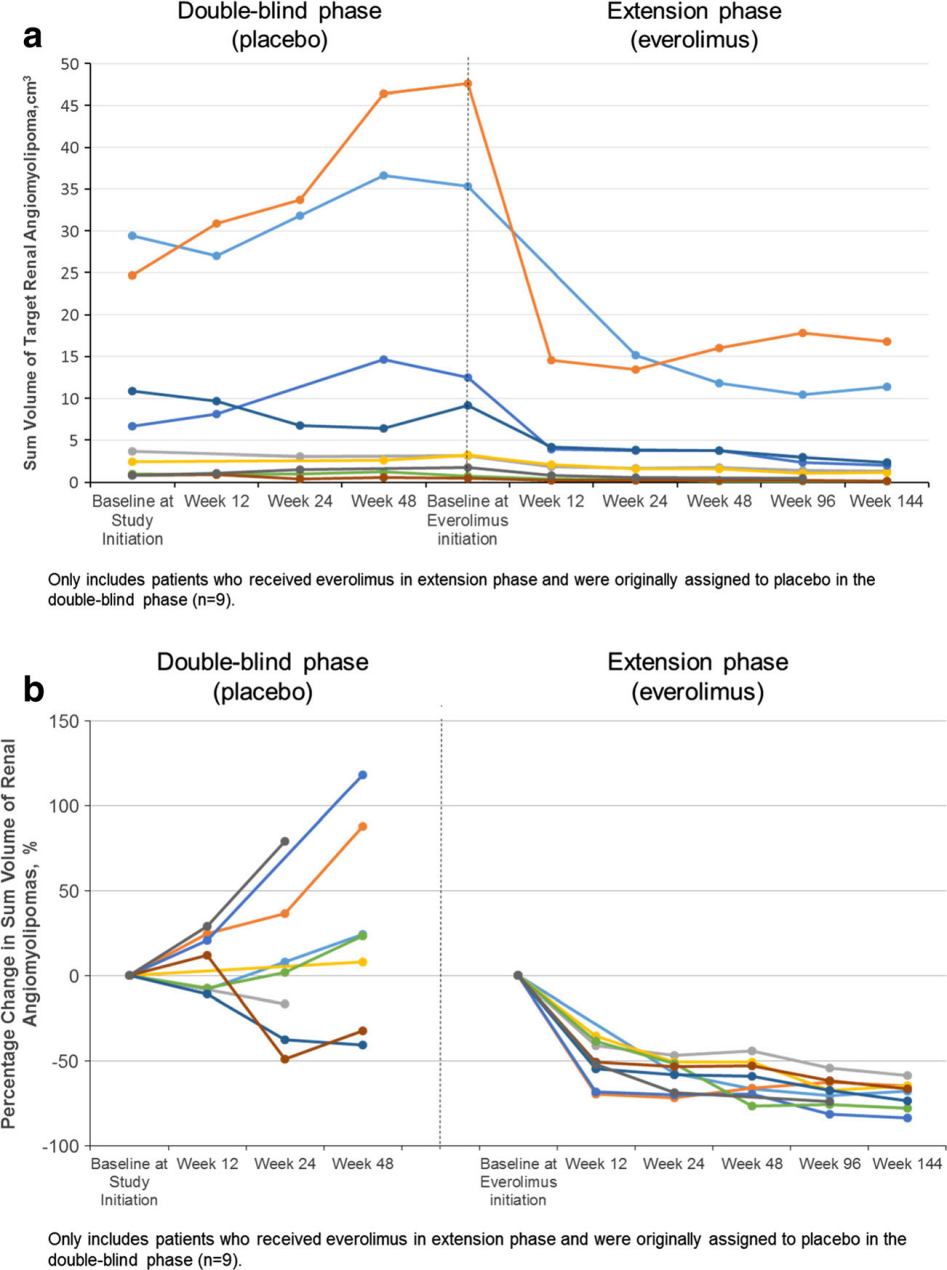
Angiomyolipoma volume over time in patients switching from placebo to everolimus, as measured by** a** the sum of volumes of the target angiomyolipoma and** b** the percentage change from baseline

Nine patients in this subgroup were randomly assigned to receive placebo during the double-blind primary core phase of the study and went on to receive everolimus in the long-term, open-label extension phase. During the placebo phase, no clear trend in angiomyolipoma volume changes from baseline was observed in these patients; however, angiomyolipoma volume decreased in all 9 patients after everolimus initiation (Fig. 3).

### Safety

All patients experienced ≥1 AE during the study, with most (*n* = 30; 90.9%) experiencing an AE that was suspected to be related to everolimus. The most commonly reported AEs of any grade occurring in more than 25% of patients included convulsion and mouth ulceration (45.5% each), stomatitis (42.4%), and cough (27.3%; Table 2). Four patients (12.1%) were reported to have hypertension. Approximately half of the patients (*n* = 18; 54.5%) experienced ≥1 grade 3 or 4 AE; 30.3% of patients experienced a grade 3 or 4 AE that was suspected to be related to everolimus. The most common grade 3 AEs (regardless of the study drug relationship) included pneumonia, convulsion, stomatitis (*n* = 3 each, 9.1%) and amenorrhea (*n* = 2 out of 10 at-risk female patients aged 10 to <18; 20%). Of the 2 cases of grade 3 amenorrhea, 1 resolved after 296 days with treatment, and 1 was ongoing at the time of the data cutoff. Grade 4 AEs (all-cause) included pyrexia, pneumonia, gastroenteritis, and hyperkalemia (*n* = 1 each; 3.0%). No cases of non-infectious pneumonitis were reported among this subgroup. All patients required additional therapy (pharmacological or nonpharmacological) to treat an AE at some point in the study.

**Table 2:** Adverse events (AEs) of any grade occurring in >15% of patients

AE	Everolimus (*n* = 33)
Any	33 (100)
Convulsion	15 (45.5)
Mouth ulceration	15 (45.5)
Stomatitis	14 (42.4)
Cough	9 (27.3)
Nasopharyngitis	8 (24.2)
Headache	7 (21.2)
Sinusitis	7 (21.2)
Upper respiratory tract infection	7 (21.2)
Blood cholesterol increase	6 (18.2)
Otitis media	6 (18.2)
Pyrexia	6 (18.2)
Vomiting	6 (18.2)
Acne	5 (15.2)
Aggression	5 (15.2)
Bronchitis	5 (15.2)
Diarrhea	5 (15.2)
Fatigue	5 (15.2)
Pneumonia	5 (15.2)
Rash	5 (15.2)
Streptococcal pharyngitis	5 (15.2)
Viral gastroenteritis	5 (15.2)

Data are presented as the number of patients with the percentage given in parenthesis

Three patients (9.1%) discontinued everolimus because of an AE. An 8.5-year-old girl discontinued treatment because of grade 3 neutropenia, which was suspected by the investigator to be everolimus-related. A 13.8-year-old boy underwent grade 3 neurosurgery because of epilepsy, which was not suspected to be everolimus-related by the investigator. A 5.9-year-old boy discontinued treatment because of grade 2 aggression brought on after experiencing grade 3 convulsion. The aggression was suspected by the investigator to be related to everolimus.

### Renal function

Overall, everolimus appeared to have no significant effect on clearance function in these patients. In general, this population had primarily normal glomerular filtration rate (GFR), with some patients having hyperfiltration. Mean (SD) GFR remained stable over the course of the stud, as shown in the Electronic Supplementary Material. None of the patients had a renal bleeding episode while on everolimus. Most patients (*n* = 26; 78.8%) also had negative protein results on urinalysis at baseline. Intermittent proteinuria occurs in TSC-related renal disease, and the level of proteinuria was exacerbated in 9 patients (27.3%) who had a protein urinalysis value of 2+ or higher at least once during the study. These measurements, however, were primarily interspersed among mostly trace or negative results. Only one patient had consistent dipstick measurements of 2+ or higher after 800 days of treatment. Proteinuria was reported as an AE in only two patients. No renal aneurysms were reported.

## Discussion

The EXIST-1 trial demonstrated the effectiveness of everolimus in reducing SEGA volume with sustained responses over approximately 4 years of treatment [23]. A preplanned secondary analysis was to evaluate the long-term efficacy and safety of everolimus in the subgroup of pediatric patients with renal angiomyolipoma. Results from this analysis demonstrate the effectiveness of everolimus in reducing renal angiomyolipoma volume in these patients, with approximately 76% of patients achieving an angiomyolipoma response. Most patients (>80%) achieved angiomyolipoma volume reductions of at least 30% over the duration of the study, and reductions of least 50% from week 24 onward, thus highlighting the sustained efficacy of everolimus for treating TSC-related renal angiomyolipoma in this pediatric subgroup. Furthermore, everolimus clearly modified the trajectory of renal angiomyolipoma growth in all pediatric patients who had originally received placebo during the double-blind phase of EXIST-1, with clear volume reductions following the initiation of open-label everolimus.

Although everolimus is currently not indicated for the treatment of renal angiomyolipoma in pediatric patients, the results in the current pediatric subpopulation are consistent with those seen in adult patients. In the EXIST-2 trial, an interim analysis after approximately 2.5 years of treatment found that everolimus was associated with a renal angiomyolipoma response rate of 54% in patients with TSC- or sporadic lymphangioleiomyomatosis-associated angiomyolipomata [18, 19]. Although a higher angiomyolipoma response rate was reported in the current analysis (76% vs 54%), it should be noted that patients in EXIST-2 had more severe disease (i.e., target angiomyolipoma >3 cm in the longest diameter in EXIST-2 vs >1 cm in EXIST-1), and the values reported in that article were after a shorter duration of time (median 28.9 vs 44.8 months). It is also possible that in EXIST-1 the measurements may not have been as precise because of the relatively smaller target lesion size, which may be more difficult to measure given the slice thickness of the imaging measure. Everolimus dosing should also be considered. Whereas EXIST-1 dosing focused on a targeted trough level (5–15 ng/mL), EXIST-2 used a starting dose of 10 mg, which could be subsequently adjusted in the event of toxicity without monitoring of the trough level [18–23].

Controlling the growth of angiomyolipomata is an important goal of treatment to avoid the development of future complications. The growth of angiomyolipomata may lead to the loss of normal renal tissue and result in the development of chronic kidney disease (CKD) and subsequent renal failure [14, 15]. The vasculature of renal angiomyolipomata is often abnormal, resulting in the development of aneurysms and increasing the risk of spontaneous hemorrhage [14]. Risk factors for angiomyolipoma-related hemorrhage include angiomyolipoma size (i.e., >3 cm), aneurysm size >0.5 cm, and serial tumor growth [13, 14, 27]. Angiomyolipoma-related renal hemorrhage is less common in the pediatric setting, but cases have been previously reported, even in children with small tumors [28–30]. Close monitoring of renal angiomyolipomata throughout life is warranted, along with early treatment to control the growth of these tumors and potentially reduce the risk for spontaneous renal hemorrhages. In this analysis, everolimus effectively reduced angiomyolipoma size, with reductions being sustained over a period of approximately 4 years. Therefore, treatment with everolimus to prevent the development of large lesions (i.e., >3 cm) in patients with serial growth or aneurysm size >0.5 cm may be a means of reducing the risk of bleeding and/or CKD among high-risk pediatric patients.

The AE profile in this subgroup of pediatric patients was generally consistent with those previously reported for everolimus in the TSC setting [18–20, 22, 31, 32]. Although all patients had ≥1 AE, with convulsion, mouth ulceration, stomatitis, and cough being the most frequently reported events, individual grade 3 or 4 events occurred in no more than 4 patients (12.1%) each, and discontinuation due to an everolimus-related AE was reported in only 2 patients (6%). As expected, AEs associated with everolimus therapy were frequently reported in this pediatric subgroup, which is consistent with the findings of higher incidence rates of mouth ulceration (32% vs 5%) and stomatitis (31% vs 21%) for everolimus versus placebo during the primary core phase of EXIST-1 [20]. Although convulsions were frequently reported in this subgroup (45.5%), no differences were observed between the everolimus and placebo arms during the primary core phase of EXIST-1 (23% vs 26%), and a similarly high incidence rate (30%) was reported in the overall population during the long-term extension phase of the study [20, 22]. However, the incidence of convulsions as a newly emergent event declined substantially over time, with 21.6% reported during the 1st year of treatment and 7% during the 5th year [23]. Other AEs that occurred with similar frequency between the everolimus and placebo arms of EXIST-1 included vomiting (17% vs 13%), cough (13% vs 10%), and nasopharyngitis (18% vs 23%) [20]. Regarding infections, incidences of individual infections in the pediatric EXIST-1 angiomyolipoma population had some variation compared with adult patients in the long-term interim analysis of the EXIST-2 study. Nasopharyngitis occurred in few patients in the EXIST-1 subgroup compared with EXIST-2 patients (24.2% vs 42.9%); however, upper respiratory tract infection (21.2% vs 14.3%) and sinusitis (21.2% vs 12.5%) occurred in slightly more patients in the EXIST-1 pediatric patient subgroup [33]. It should also be noted that nearly all patients (99%) required additional therapy (drug or nondrug) to treat an AE at some point in the study. This should be taken into consideration, as the addition of more therapies may have an impact on the patient’s quality of life.

Although a deeper analysis into the effects of everolimus on growth and sexual maturation were beyond the scope of this secondary analysis, related AEs such as amenorrhea were observed in 2 out of 10 at-risk female patients. This rate is similar to that found in the entire EXIST-1 cohort [23]. Even though the event in one patient of this subgroup was ongoing at study end, in general, most cases of amenorrhea tended to resolve either spontaneously without intervention or with dose modification (reduction or temporary dose interruption). A more detailed analysis of the effects of everolimus on sexual maturation and menstrual irregularities using pooled data from three major studies (EXIST-1, EXIST-2, phase 2 study in SEGA) is currently in development. With regard to patient growth, in previous reports of the entire population of pediatric patients from EXIST-1, no differences in SD scores were seen in height and weight before and during treatment with everolimus [22, 23].

There appeared to be no appreciable change in renal function based on mean calculated GFR in this pediatric subgroup. The interim analysis of EXIST-2 also showed a similar finding in adult patients with angiomyolipoma treated with everolimus, with no significant decline in mean GFR over time. However, it should be noted that the mean GFR at baseline in EXIST-2 appeared to be lower than that of pediatric patients in the current analysis, but this observation is most likely attributed to the use of different methods to estimate GFR, and higher patient age and more advanced disease in EXIST-2. The majority of patients in the current analysis from EXIST-1 had mostly negative or trace protein results on urinalysis, with proteinuria being reported as an AE in only 2 patients, and persistently elevated urinary protein levels occurring in 1 patient. It should also be noted that some patients in the study were taking antiepileptic medications, including topiramate. Topiramate has been shown to induce metabolic acidosis accompanied by an alkaline urine [34]. Alkaline urine (pH >7.5) can lead to false-positive results for proteinuria on urine dipstick tests [35]. However, to ensure safety, proteinuria should be monitored in everolimus-treated patients.

This analysis of EXIST-1 is associated with a number of limitations that should be considered. First, the open-label design of the long-term extension phase and the lack of a comparator arm may introduce bias and limit the ability to draw overall conclusions on the long-term use of everolimus. Additionally, the analysis was not adequately powered to assess this subgroup, because of the small sample size of this subset of pediatric patients with angiomyolipoma. Furthermore, although urine dipstick assessment is an acceptable method of detecting elevated urinary protein levels, the presence of proteinuria should be confirmed by a quantitative measurement, such as the urine protein-to-creatinine ratio. Last, there is an inherent risk of measurement errors in smaller lesions. The abdominal MRI performed used 5-mm cuts, and at baseline lesions of 1 cm would only have two possible cuts for evaluation. As such lesions shrink, the accuracy of measurement decreases, even though the shrinkage is real.

## Conclusions

Although everolimus is currently not indicated for use in treating renal angiomyolipoma in pediatric patients, in this pediatric population with renal angiomyolipoma being treated for TSC-associated SEGA, renal angiomyolipoma volumes were reduced, and these reductions were maintained over the approximately 4-year study. Observed AEs were consistent with the known safety profile of everolimus in TSC, and no appreciable effect on renal function was observed. Therefore, these findings support the efficacy and safety of everolimus in treating pediatric patients with renal angiomyolipoma and SEGA requiring everolimus treatment.

## Electronic supplementary material

ESM 1 (DOCX 50 kb)

## References

[1] Franz DN, Bissler JJ, McCormack FX (2010). Tuberous sclerosis complex: neurological, renal and pulmonary manifestations. Neuropediatrics.

[2] Osborne JP, Fryer A, Webb D (1991). Epidemiology of tuberous sclerosis. Ann N Y Acad Sci.

[3] Huang J, Manning BD (2008). The TSC1-TSC2 complex: a molecular switchboard controlling cell growth. Biochem J.

[4] Borkowska J, Schwartz RA, Kotulska K, Jozwiak S (2011). Tuberous sclerosis complex: tumors and tumorigenesis. Int J Dermatol.

[5] Jozwiak S, Kotulska K, Kasprzyk-Obara J, Domanska-Pakiela D, Tomyn-Drabik M, Roberts P, Kwiatkowski D (2006). Clinical and genotype studies of cardiac tumors in 154 patients with tuberous sclerosis complex. Pediatrics.

[6] Isaacs H (2009). Perinatal (fetal and neonatal) tuberous sclerosis: a review. Am J Perinatol.

[7] Goh S, Butler W, Thiele EA (2004). Subependymal giant cell tumors in tuberous sclerosis complex. Neurology.

[8] Adriaensen ME, Schaefer-Prokop CM, Stijnen T, Duyndam DA, Zonnenberg BA, Prokop M (2009). Prevalence of subependymal giant cell tumors in patients with tuberous sclerosis and a review of the literature. Eur J Neurol.

[9] Budde K, Gaedeke J (2012). Tuberous sclerosis complex-associated angiomyolipomas: focus on mTOR inhibition. Am J Kidney Dis.

[10] Crino PB, Nathanson KL, Henske EP (2006). The tuberous sclerosis complex. N Engl J Med.

[11] Rakowski SK, Winterkorn EB, Paul E, Steele DJ, Halpern EF, Thiele EA (2006). Renal manifestations of tuberous sclerosis complex: incidence, prognosis, and predictive factors. Kidney Int.

[12] Tsai JD, Wei CC, Chen SM, Lue KH, Sheu JN (2014). Association between the growth rate of renal cysts/angiomyolipomas and age in the patients with tuberous sclerosis complex. Int Urol Nephrol.

[13] Kingswood JC, Doyle T, Cox J, Mbundi J, Attard V, Patel U, Saggar A, Elmslie F (2012). The natural history of renal angiomyolipomata (AMLS) in tuberous sclerosis complex (TSC). Nephrol Dial Transplant.

[14] Pirson Y (2013). Tuberous sclerosis complex-associated kidney angiomyolipoma: from contemplation to action. Nephrol Dial Transplant.

[15] Bissler JJ, Kingswood JC (2004). Renal angiomyolipomata. Kidney Int.

[16] Shepherd CW, Gomez MR, Lie JT, Crowson CS (1991). Causes of death in patients with tuberous sclerosis. Mayo Clin Proc.

[17] Eijkemans MJ, van der Wal W, Reijnders LJ, Roes KC, van Waalwijk van Doorn-Khosrovani SB, Pelletier C, Magestro M, Zonnenberg B (2015). Long-term follow-up assessing renal angiomyolipoma treatment patterns, morbidity, and mortality: an observational study in tuberous sclerosis complex patients in the Netherlands. Am J Kidney Dis.

[18] Bissler JJ, Kingswood JC, Radzikowska E, Zonnenberg BA, Frost M, Belousova E, Sauter M, Nonomura N, Brakemeier S, de Vries PJ, Whittemore VH, Chen D, Sahmoud T, Shah G, Lincy J, Lebwohl D, Budde K (2013). Everolimus for angiomyolipoma associated with tuberous sclerosis complex or sporadic lymphangioleiomyomatosis (EXIST-2): a multicentre, randomised, double-blind, placebo-controlled trial. Lancet.

[19] Bissler JJ, Kingswood JC, Radzikowska E, Zonnenberg BA, Frost M, Belousova E, Sauter M, Nonomura N, Brakemeier S, de Vries PJ, Berkowitz N, Miao S, Segal S, Peyrard S, Budde K (2015). Everolimus for renal angiomyolipoma in patients with tuberous sclerosis complex or sporadic lymphangioleiomyomatosis: extension of a randomized controlled trial. Nephrol Dial Transplant.

[20] Franz DN, Belousova E, Sparagana S, Bebin EM, Frost M, Kuperman R, Witt O, Kohrman MH, Flamini JR, Wu JY, Curatolo P, de Vries PJ, Whittemore VH, Thiele EA, Ford JP, Shah G, Cauwel H, Lebwohl D, Sahmoud T, Jozwiak S (2013). Efficacy and safety of everolimus for subependymal giant cell astrocytomas associated with tuberous sclerosis complex (EXIST-1): a multicentre, randomised, placebo-controlled phase 3 trial. Lancet.

[21] Novartis Pharmaceuticals Corporation (2015). Afinitor [package insert].

[22] Franz DN, Belousova E, Sparagana S, Bebin EM, Frost M, Kuperman R, Witt O, Kohrman MH, Flamini JR, Wu JY, Curatolo P, de Vries PJ, Berkowitz N, Anak O, Nilat J, Jozwiak S (2014). Everolimus for subependymal giant cell astrocytoma in patients with tuberous sclerosis complex: 2-year open-label extension of the randomised EXIST-1 study. Lancet Oncol.

[23] Franz DN, Belousova E, Sparagana S, Bebin EM, Frost MD, Kuperman R, Witt O, Kohrman MH, Flamini JR, Wu JY, Curatolo P, de Vries PJ, Berkowitz N, Niolat J, Jozwiak S (2016). Long-term use of everolimus in patients with tuberous sclerosis complex: final results from the EXIST-1 study. PLoS One.

[24] Kingswood JC, Jozwiak S, Belousova ED, Frost MD, Kuperman RA, Bebin EM, Korf BR, Flamini JR, Kohrman MH, Sparagana SP, Wu JY, Brechenmacher T, Stein K, Berkowitz N, Bissler JJ, Franz DN (2014). The effect of everolimus on renal angiomyolipoma in patients with tuberous sclerosis complex being treated for subependymal giant cell astrocytoma: subgroup results from the randomized, placebo-controlled, phase 3 trial EXIST-1. Nephrol Dial Transplant.

[25] National Cancer Institute (2006). Cancer Therapy Evaluation Program: Common Terminology Criteria for Adverse Events v3.0 (CTCAE).

[26] Schwartz GJ, Munoz A, Schneider MF, Mak RH, Kaskel F, Warady BA, Furth SL (2009). New equations to estimate GFR in children with CKD. J Am Soc Nephrol.

[27] Kingswood JC, Doyle T, Cox J, Mbundi J, Attard V, Patel U, Saggar A, Elmslie F (2013). The long term outcome or renal angiomyolipomata (AMLs) in tuberous sclerosis complex (TSC). Dev Med Child Neurol.

[28] Kingswood C, Bolton P, Crawford P, Harland C, Johnson SR, Sampson JR, Shepherd C, Spink J, Demuth D, Lucchese L, Nasuti P, Gray E, Pinnegar A, Magestro M (2016). The clinical profile of tuberous sclerosis complex (TSC) in the United Kingdom: a retrospective cohort study in the Clinical Practice Research Datalink (CPRD). Eur J Paediatr Neurol.

[29] Avolio L, Savasta S, Matteotti C, Fusillo M, Bragheri R (2001). Symptomatic unilateral renal angiomyolipoma in a child with tuberous sclerosis. Urol Int.

[30] O'Callaghan FJ, Noakes MJ, Martyn CN, Osborne JP (2004). An epidemiological study of renal pathology in tuberous sclerosis complex. BJU Int.

[31] Krueger DA, Care MM, Holland K, Agricola K, Tudor C, Mangeshkar P, Wilson KA, Byars A, Sahmoud T, Franz DN (2010). Everolimus for subependymal giant-cell astrocytomas in tuberous sclerosis. N Engl J Med.

[32] Krueger DA, Care MM, Agricola K, Tudor C, Mays M, Franz DN (2013). Everolimus long-term safety and efficacy in subependymal giant-cell astrocytoma. Neurology.

[33] Bissler JJ, Kingswood JC, Radzikowska E, Zonnenberg BA, Frost M, Belousova E, Sauter M, Nonomura N, Brakemeier S, de Vries PJ, Berkowitz N, Miao S, Segal S, Peyrard S, Budde K (2016). Everolimus for renal angiomyolipoma in patients with tuberous sclerosis complex or sporadic lymphangioleiomyomatosis: extension of a randomized controlled trial. Nephrol Dial Transplant.

[34] Mirza N, Marson AG, Pirmohamed M (2009). Effect of topiramate on acid-base balance: extent, mechanism and effects. Br J Clin Pharmacol.

[35] Carroll MF, Temte JL (2000). Proteinuria in adults: a diagnostic approach. Am Fam Physician.

