# Supplementary material for: The effect of everolimus on renal angiomyolipoma in pediatric patients with tuberous sclerosis being treated for subependymal giant cell astrocytoma
Source: Pediatr Nephrol. 2017 Oct 9;33(1):101–9. doi: 10.1007/s00467-017-3806-1 (PMC5700234; doi:10.1007/s00467-017-3806-1)
Supplement: Supplementary file 1 — (DOCX 50 kb) [file 467_2017_3806_MOESM1_ESM.docx]

**The effect of everolimus on renal angiomyolipoma in pediatric patients with tuberous sclerosis being treated for subependymal giant cell astrocytoma**

**Journal**: *Pediatric Nephrology*

John J. Bissler, David N. Franz, Michael D. Frost, Elena Belousova, E. Martina Bebin, Steven Sparagana, Noah Berkowitz, Antonia Ridolfi, J. Christopher Kingswood

**Corresponding author:**

(J. Chris Kingswood)

Royal Sussex County Hospital, Brighton, UK

E-mail: [Chris.Kingswood@bsuh.nhs.uk](mailto:Chris.Kingswood@bsuh.nhs.uk)

**Electronic Supplementary Material**

**Online Resource 1** Mean (standard deviation [SD]) glomerular filtration rate (GFR) over time in the pediatric renal angiomyolipoma population


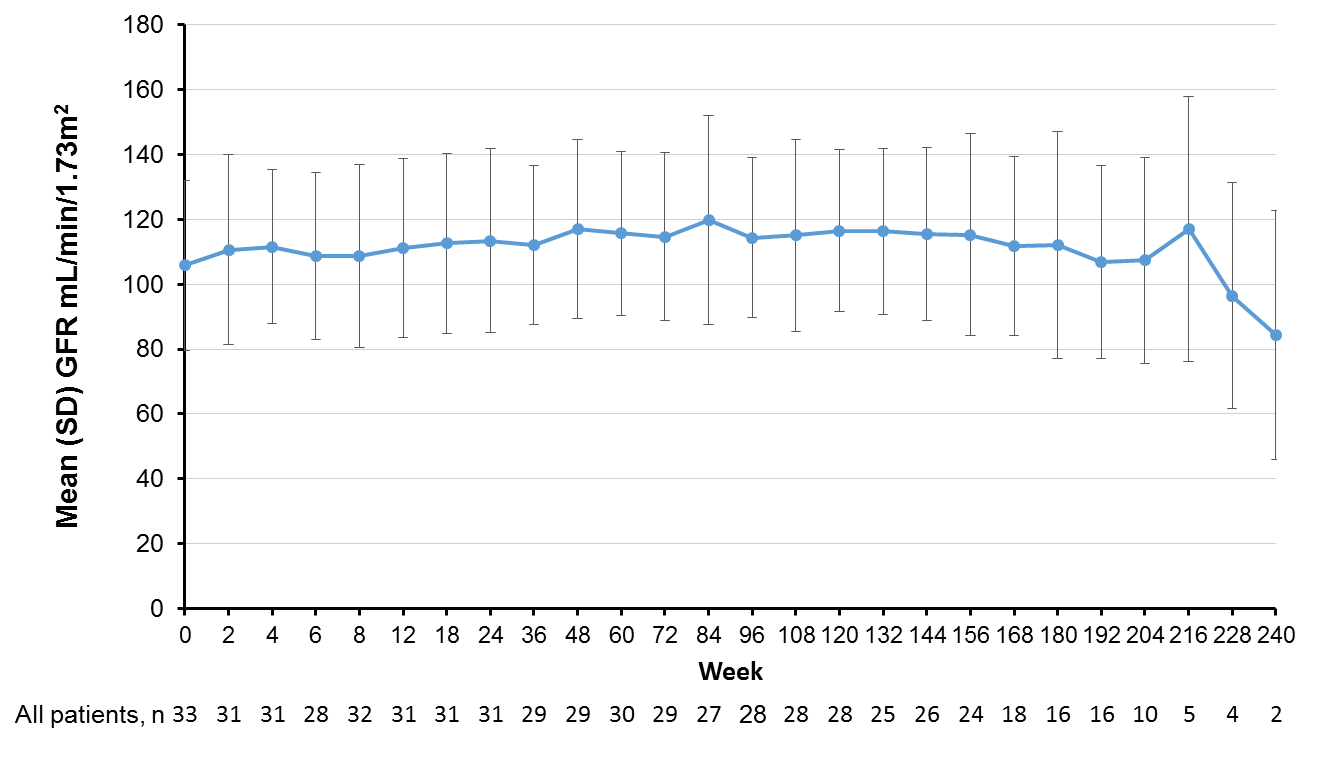


**Online Resource 2**. List of independent ethics committees (IEC) or institutional review boards (IRB) by study center

| **Center No.** | **Ethics Committee or**  **Institutional Review Board** | **Department /**  **Organization** | **City, State/Province,**  **Postal Code**  **Country** |
| --- | --- | --- | --- |
| 0100 | SESIAHS Human Research  Ethics Committee | SESIAHS Northern  Hospital Network -  Research Office | Randwick NSW 2130,  Australia |
| 0150 | Commissie voor Medische Ethiek | Universitair Ziekenhuis Brussel | Brussel 1090, Belgium |
| 0250 | Research Ethics Board | The Sick Childrens Hospital | Toronto M5G 1X8, Canada |
| 0251 | Comité d'éthique de recherche | Hôpital Saint Justine | Montreal Quebec H3T,  IC5  Canada |
| 0352 | Ethik-Kommission | Medizinischen Falultät Heidelberg | Heidelberg 69115,  Germany |
| 0353 | Geschäftsstelle der EthikKommission des Landes Berlin | Landesamt für  Gesundheit und Soziales | Berlin 10707,  Germany |
| 0400 | Comitato Etico Indipendente | Azienda Ospedaliera  Universitaria Policlinico  Tor Vergata di Roma | Roma 00133,  Italy |
| 0403 | Comitato Di Etica | IRCSS Istituto Giannina Gaslini di Genova | Genova GE 16147, Italy |
| 0450 | Medische Ethische Toetsingscommissie | Universitair Medisch  Centrum Utrecht | Utrecht 3508 GA,  Netherlands |
| 0600 | Komisja Bioetyczna | Instytucie "Pomnik  Zdrowia Dziecka" | Warszawa 04-730,  Poland |
| 0700 | Ethics Committee | Moscow research institute of pediatrics and children surgery, Ministry of Health of Russia | Moscow NA 125412, Russia |
| 0753 | Southampton and South West Hampshire Research Ethics  Committee | NHS - Health Research Authority | Southampton Hampshire  SO16 4RJ,  United Kingdom |
| 0500 | Institutional Review Board | Cincinnati Children's  Hospital Medical center | Cincinnati MLC 5020, United States |
| 0501 | Western Institutional Review Board | WIRB - Copernicus Group | Olympia WA 98502, United States |
| 0502 | Western Institutional Review Board | WIRB - Copernicus Group | Olympia WA 98502, United States |
| 0503 | 1. Adult consent: Institutional Review Board 2. Children consent: Institutional Review Board | 1. Allina Health System 2. Children's Hospitals and Clinics of Minnesota | Minneapolis MN 55404, United States  Minneapolis MN 55404, United States |
| 0504 | Partners Human Research office | Partners HealthCare | Boston MA 02116, United States |
| 0509 | Institutional Review Board | The University of Chicago | Chicago IL 60637, United States |
| 0510 | Institutional Review Board | The University of Texas, Southwestern medical center | Dallas TX 75390-8843,  United States |
| 0511 | Western Institutional Review Board | WIRB - Copernicus Group | Olympia WA 98502, United States |
| 0512 | Office of Human Research Studies | Dana Farber Cancer Institute | Boston MA 02115, United States |
| 0514 | Internal Review Board | St. Joseph's Hospital and Medical Center | Phoenix AZ 85013, United States |
| 0515 | Institutional Review Board | Children's Hospital and Research Center Oakland | Oakland CA 94609 United States |
| 0516 | Institutional Review Board | Children's Healthcare of Atlanta | Atlanta GA 30329, United States |
